# Editorial: Cardiovascular inflammaging: basic and translational aspects

**DOI:** 10.3389/fcvm.2024.1385683

**Published:** 2024-03-01

**Authors:** Maria Luisa Barcena, Muhammad Aslam, Yury Ladilov

**Affiliations:** ^1^ Department of Geriatrics and Medical Gerontology, Charité –Universitätsmedizin Berlin, Corporate Member of Freie Universität Berlin, Humboldt-Universität zu Berlin, and Berlin Institute of Health, Berlin, Germany; ^2^ DZHK (German Centre for Cardiovascular Research, Partner Side Berlin), Berlin, Germany; ^3^ Experimental Cardiology, Department of Internal Medicine I, Justus Liebig University, Giessen, Germany; ^4^ Department of Cardiology, Kerckhoff Clinic GmbH, Bad Nauheim, Germany; ^5^ DZHK (German Centre for Cardiovascular Research, Partner Side Rhine/Main, Bad Nauheim/Frankfurt/Mainz), Bad Nauheim, Germany; ^6^ Department of Cardiovascular Surgery, Heart Center Brandenburg, Brandenburg Medical School, Bernau bei Berlin, Germany

**Keywords:** inflammaging, translational aspects, cardiovascular disease, inflammation, mtDNA

**Editorial on the Research Topic** Cardiovascular inflammaging: basic and translational aspects

Growing evidence indicates that chronic systemic inflammation, commonly referred to as “inflammaging” is an important feature of aging. The underlying cellular mechanisms are complex and involve multifactorial processes, e.g., oxidative stress, mitochondrial dysfunction, accumulation of senescent cells, impaired autophagy, and alterations in the microbiome (1–3).

Chronic systemic inflammation, even at a low rate, can result in organ damage and may contribute to the development of various diseases including cardiovascular diseases (CVD) (4). Conversely, several comorbidities, such as obesity, diabetes, and hypertension, can promote systemic inflammation (5).

Moreover, inflammation is frequently observed in the aging vasculature and the heart. Specifically, vascular aging is linked to more severe atherosclerosis and microvascular dysfunction, characterized by pathological vascular remodeling and vascular stiffness (6). Inflammation is also associated with the development of heart failure with preserved ejection fraction (HFpEF). Importantly, this type of heart failure is significantly more prevalent in elderly women (7), potentially attributable to estrogen loss or still poorly understood intrinsic cellular sex differences.

The current topic offers novel perspectives and insights into the inflammatory processes associated with aging in the cardiovascular system, encompassing underlying cellular mechanisms and protective strategies. In this editorial, we present a succinct overview of recently published original research and review articles that contribute to our understanding of how aging impacts inflammation in the cardiovascular system.

The study conducted by Zhai et al. explored the impact of aging on myocardial inflammation in response to sepsis, utilizing a mouse model involving cecal ligation and puncture. The induction of sepsis led to an elevated mortality rate of 50% in older male mice, whereas younger mice exhibited survival. The increased mortality appeared to be closely linked to sepsis-induced cardiac dysfunction in older mice, as evidenced by a reduced ejection fraction (EF: 18%) and cardiac output (2.4 ml/min) observed two days after ligation. Indeed, the mortality rate in older septic mice showed a direct correlation with cardiac function. The compromised cardiac function was accompanied by an exacerbated expression of prominent pro-inflammatory cytokines, such as TNF-α, IL-1β, IL-6, and MCP-1, in the myocardium and plasma specifically in older septic mice. Moreover, the authors identified that toll-like receptor 2 (TLR2) was upregulated in the myocardium of older mice but not in younger mice. The genetic ablation of TLR2 resulted in improved cardiac function and diminished production of pro-inflammatory cytokines, reinforcing the role of TLR2 in the sepsis-related cardiac dysfunction in older mice. Hence, modulation of the TLR2 expression/activity emerges as a potentially crucial approach to enhance cardiac function and decrease mortality in elderly septic patients.

Due to its intrinsic role as a potent anti-inflammatory cytokine, erythropoietin (EPO) is under investigation as a potential therapeutic for cardiovascular diseases (8). However, the erythropoiesis stimulated by recombinant EPO therapy often outweighs the anti-inflammatory cytoprotective effects. To address this issue, researchers have explored the extra-hematopoietic cytoprotective effects of EPO, which can be achieved by a small peptide derivative known as ARA290, lacking hematopoietic effects (9, 10). In the study by Winicki et al., the effects of ARA290 (cibinetide) administration, a specific agonist of erythropoietin/CD131 heteroreceptor, were investigated on cardiac function, cardiac inflammation, and mitochondrial function in cardiomyocytes from older Fischer 344 × Brown Norway rats. The authors demonstrated that the chronic ARA290 administration diminished age-associated impaired cardiac function and cardiac inflammation. Moreover, it reduced the number of pro-inflammatory leukocytes and monocytes, improved mitochondrial proteostasis, and reduced systemic frailty in older rats. Additionally, cardiomyocytes from treated rats exhibited higher thresholds for mitochondrial permeability transition pore (mPTP) opening induced by reactive oxygen species (ROS), and autophagy flux. Overall, the authors suggest an ARA290-dependent decline in systemic inflammation, leading to improved left ventricular systolic function and preserved late-age body weight. Consequently, a decrease in frailty and an improvement in the health span were observed in these advanced-aged rats.

In another study, Headley and Tsao conducted a review of the literature on age-related mitochondrial dysfunction and briefly introduced a developing therapeutic area. With a focus on mitochondrial dysfunction in vascular aging, the authors emphasize the complexity of aging-related alterations in mitochondrial biology. Specifically, they highlighted the decline in mitochondrial function and biogenesis, as well as the impairment of mitophagy, increased mitochondrial ROS formation, and mitochondrial DNA (mtDNA) damage, particularly in endothelial and smooth muscle cells. Of note, mtDNA is located within the mitochondrial matrix and, thus, continuously exposed to the ROS generated inside mitochondria. The authors highlighted the importance of mtDNA escaping from mitochondria into the cytosol, triggering inflammatory responses via TLR9, cGas-STING, and NLRP3 inflammasome signaling. Chronic activation of these cellular responses contributes to aging-associated inflammation. Furthermore, the authors discussed various aspects of modulating mitochondrial dysfunction, with a particular focus on highly debated mitochondrial transplantation. They explored methodological and clinical considerations of mitochondrial transplantation and discussed recent advances in this therapeutic approach. However, readers are cautioned about the controversies and doubts surrounding the protective mechanisms and clinical implication of the technique (11).

Lastly, Hua et al. conducted a comprehensive review of the current literature on vascular fibrosis and its implications in the development and progression of cardiovascular disorders. They discuss various molecules and signaling pathways including renin-angiotensin system, TGF-β signaling, IL-11 signaling, inflammation, and mitochondrial oxidative stress, which play roles in vascular fibrosis. Additionally, the authors discuss the effects of ageing as well as sex-related differences in ageing-mediated vascular fibrosis. Furthermore, the review delves into potential molecular targets for therapeutic intervention in vascular fibrosis to effectively treat CVD.

In summary, this research topic explores some actual trends in both basic and translational research in the area of cardiovascular inflammaging, highlighting its significance in the aging process and its implications for cardiovascular health. The topic provides insights into the cellular mechanisms underlying inflammaging and explores potential therapeutic strategies. The study investigating the impact of aging on myocardial inflammation in response to sepsis reveals promising avenues for targeting TLR2 to improve cardiac function and reduce mortality in elderly septic patients. Additionally, EPO-derived peptide is being investigated as a potential therapeutic for CVD due to its anti-inflammatory properties. An important area of focus is mitochondrial dysfunction in vascular aging, with mitochondrial transplantation emerging as a potentially beneficial therapeutic approach. The topic also discusses vascular fibrosis and its role in cardiovascular disorders, highlighting molecular targets for therapeutic intervention.
